# *In vitro* inhibition of acetylcholinesterase activity by yellow field pea (*Pisum sativum*) protein-derived peptides as revealed by kinetics and molecular docking

**DOI:** 10.3389/fnut.2022.1021893

**Published:** 2022-10-21

**Authors:** Nancy D. Asen, Ogadimma D. Okagu, Chibuike C. Udenigwe, Rotimi E. Aluko

**Affiliations:** ^1^Department of Food and Human Nutritional Sciences, University of Manitoba, Winnipeg, MB, Canada; ^2^Department of Chemistry and Biomolecular Sciences, Faculty of Science, University of Ottawa, Ottawa, ON, Canada; ^3^School of Nutrition Sciences, Faculty of Health Sciences, University of Ottawa, Ottawa, ON, Canada; ^4^Richardson Centre for Food Technology and Research, University of Manitoba, Winnipeg, MB, Canada

**Keywords:** acetylcholinesterase, acetylcholine, peptide, inhibition, interaction, kinetics, molecular docking, Alzheimer’s disease

## Abstract

Compounds with structural similarities to the neurotransmitter (acetylcholine) are mostly used to inhibit the activity of acetylcholinesterase (AChE) in Alzheimer’s disease (AD) therapy. However, the existing drugs only alleviate symptoms of moderate to mild conditions and come with side effects; hence, the search is still on for potent and safer options. In this study, High performance liquid chromatography (HPLC) fractionations of AChE-inhibitory pea protein hydrolysates obtained from alcalase, flavourzyme and pepsin digestions were carried out followed by sequence identification of the most active fractions using mass spectrometry. Subsequently, 20 novel peptide sequences identified from the active fractions were synthesized and five peptides, QSQS, LQHNA, SQSRS, ETRSQ, PQDER (IC_50_ = 1.53 – 1.61 μg/mL) were selected and analyzed for ability to change AChE protein conformation (fluorescence emission and circular dichroism), kinetics of enzyme inhibition, and enzyme-ligand binding configurations using molecular docking. The kinetics studies revealed different inhibition modes by the peptides with relatively low (<0.02 mM and <0.1 mM) inhibition constant and Michaelis constant, respectively, while maximum velocity was reduced. Conformational changes were confirmed by losses in fluorescence intensity and reduced α-helix content of AChE after interactions with different peptides. Molecular docking revealed binding of the peptides to both the catalytic anionic site and the peripheral anionic site. The five analyzed peptides all contained glutamine (Q) but sequences with Q in the penultimate N-terminal position (LQHNA, SQSRS, and PQDER) had stronger binding affinity. Results from the different analysis in this study confirm that the peptides obtained from enzymatic digestion of pea protein possess the potential to be used as novel AChE-inhibitory agents in AD management.

## Introduction

Acetylcholinesterase (AChE) belongs to the family of α/β serine hydrolases and plays a significant physiological role in maintaining homeostasis of neurotransmission by continual termination of neuronal signals through the breakdown of acetylcholine to acetic acid and choline (1). The neuronal cholinergic pathway comprises of acetylcholine (ACh), acetylcholine transferase (synthesizing enzyme), vesicular acetylcholine transporter, and the receptors (2). These factors together play the important role of regulating metabolic activities and cognitive abilities (i.e., learning and memory, motor, visual and spatial functions). In the early 70s, the loss of basal forebrain cholinergic neurons causing memory dysfunction in Alzheimer’s disease (AD) patients was first discovered (3). The current AD therapy involves the use of compounds with structural similarity (i.e., presence of ester bonds) to the natural substrate (ACh) to inhibit AChE activity (4, 5). Inhibition of AChE activity is important because the cholinergic pathway is one channel of interest in studying the onset and development of AD. Current therapy involves the use of drugs (tacrine, donepezil, rivastigmine, and galantamine) but this has not been very effective as only the symptoms are alleviated in mild to moderate cases while the occurrence of several cases of negative side effects have been reported (6).

To develop new therapeutic agents, understanding the structural conformation of AChE is an important information to enable better explanation of interactions with the natural substrate or ligands. AChE has a complex ellipsoid structure with dimensions of ∼45 Å by 60 Å by 65 Å and a large twisted β-sheet running throughout the structure (6). Halfway through the structure is a 20 Å deep and 5 Å wide gorge lined with 14 aromatic amino acids, which houses different domains of the active site. The main site is the esteratic or catalytic triad (Ser200, His440, and Glu327), which is buried in the gorge and the peripheral anionic site, PAS (Tyr70, Asp72, Tyr121, Trp279, and Tyr334) located at the entrance of the gorge. Allosteric binding of some inhibitors and non-cholinergic inhibition like interactions with β-amyloid occurs at the PAS (5) and this site is responsible for the initial recognition of positively charged substrates. Other subsites are the catalytic anionic site, CAS (Trp84, Tyr130, Phe330, and Phe331, oxyanion hole (Gly121, Gly122, and Ala204), acyl binding pocket (Trp236, Phe295, Phe297, and Phe338) responsible for substrate specificity, and other residues of the omega loop (Thr83, Asn87, and Pro88) (6). The CAS is located at the bottom of the gorge and binds to the quaternary ammonium group of the substrate through cation-π interactions, which maintains proper orientation of acetylcholine in the gorge (6).

Several studies have been carried out to explain the activity of cholinesterases and develop potent inhibitors (1, 4, 7–9). Kinetic studies and molecular docking are analytical tools that give information about the catalytic parameters of an enzyme and reveal key elements in the enzyme-ligand complex necessary for drug design, respectively. Kinetic studies reveal the inhibition mode and strength (affinity) of an inhibitor (i.e., competitive, non- competitive, uncompetitive, or mixed model) using factors such as maximum reaction rate (V_max_), inhibition constant (K*_i_*), and Michaelis constant (K*_m_*). The kinetics of AChE inhibition was previously carried out using secondary metabolites from an edible mushroom, *Suillus luteus* (L.), and a non-competitive mixed mechanism was reported (10). Another study carried out the kinetics of newly designed analogs of donepezil and the results revealed that the most potent AChE inhibitors exhibited a mixed model of inhibition (11). Only a few studies have reported findings from kinetics of AChE inhibition activity by peptide-containing materials such as enzymatic hydrolysates. For example, Malomo and Aluko (9) reported a mixed-type inhibition by hemp seed protein hydrolysate while Zhao et al. (12) suggested that the high lysine content in anchovy protein hydrolysates influenced competitive and non-competitive inhibitions of AChE activity. Molecular docking of interactions between AChE and potential inhibitors have been widely reported for non-peptide inhibitors. For example, Karunakaran et al. (13) studied human AChE-inhibitory activity of *Convolvulus pluricaulis* (an herb) in zebrafish with scopolamine-induced cognitive dysfunction and result showed that the inhibitor was bound to His447 of the catalytic triad, anionic subsite, and peripheral anionic site. Koca et al. (14) synthesized a benzamide derivative to investigate potency against cholinesterases with predicted inhibition profiles of the derivative. Furthermore, Makarian (11) analyzed the most potent derivatives of donepezil using molecular docking analysis and results showed different moieties of the new compound binding to all the subsites of the binding site. The literature reports show that information from kinetic studies and molecular docking analysis of AChE-ligand interactions is dependent on inhibitor type.

In contrast to other natural products, information from the molecular docking studies of AChE – peptide complexes are scanty. Malta et al. (15) reported molecular docking analysis of the interactions between Kefir’s bioactive peptides and AD-like flies’ model. The three-dimensional structure and molecular docking were predicted using nine peptides and result showed that peptide VYPFPGPIPN was the best ligand for human AChE binding at the PAS. Literature is replete with studies on the use of bioactive peptides derived from different sources for *in vitro* and *in vivo* inhibitory activities of other enzymes of physiological interest, such as ACE (16), renin (17), amylase and glucosidase (18). In our previous study, we reported the production of enzymatic protein hydrolysates of yellow field pea and their potency against AChE (4). In the current work, the protein hydrolysates were fractionated by reverse-phase HPLC followed by mass spectrometry analysis to identify the amino acid sequence of peptides present in the most potent AChE-inhibitory fractions. These peptides were synthesized and studied for kinetics of AChE inhibition, conformational changes of the enzyme in the presence of each peptide, and molecular interactions involved in maintaining the enzyme-peptide complexes.

## Materials and methods

### Materials

The starting material was yellow field pea protein concentrate (PPC) obtained from Nutri-Pea Limited (Portage La Prairie, MB, Canada). AChE from electric eel (16.4 units/mg protein), acetylcholine iodide (ATCI) and dithio-bis-nitrobenzoic acid (DTNB) were purchased from Sigma-Aldrich (St. Louis, MO, USA). The *de novo* synthesis of the 20 peptides (> 95% purity) was carried out by GenScript Inc. (Piscataway, NJ, USA). All chemicals and reagents were of high purity analytical grade and double distilled water (Millipore) was used in the preparation of all reagents.

### Reversed-phase high-performance liquid chromatography (RP-HPLC) fractionation of pea protein hydrolysates

The production and AChE-inhibitory properties of pea protein hydrolysates have been previously reported (4). Briefly, aqueous PPC mixtures (10%, w/v) were prepared and digested with each of alcalase (4%, w/w of substrate protein), flavourzyme (4%, w/w of substrate protein) and pepsin (1%, w/w of substrate protein). The protein digests were centrifuged and the supernatant containing soluble peptides were isolated and freeze-dried to produce hydrolysates labeled ACH, FZH, and PEH, respectively). The three hydrolysates were individually separated using a Shimadzu High Performance Liquid Chromatography (Shimadzu Scientific Instruments Inc. MD, USA) coupled with a Photodiode Array UV-VIS Detector and an Autosampler according to a protocol previously described by Sonklin et al. (16). Briefly, the respective digests (50 mg/mL) were diluted in mobile phase A (0.1% aqueous trifluoroacetic acid, TFA) and then filtered sequentially through a 0.45 μm and then 0.22 μm using a Steriflip vacuum-driven filtration system (EMD Millipore Corporation, MA, USA). Subsequently, the filtered sample (4 mL) was injected onto a 21 × 250 mm (5 μm) C12 preparative column (Phenomenex Inc., Torrance, CA, USA). The peptides were eluted using mobile phase B (0.1% TFA in methanol) at a flow rate of 10 mL/min and a linear gradient from 0 to 100% Buffer B over 60 min. The eluted peptides were detected at 214 nm and several fractions (F) of interest collected for each digest: ACH (F1-F7), FZH (F1-F7) and PEH (F1-F8). Each collected fraction was pooled and evaporated under vacuum in a rotary evaporator at 50*^o^*C to remove excess solvent and the aqueous residues were freeze dried and stored at 20*^o^*C. The initial AChE inhibition activity of the peptides was analyzed and the most potent fractions with high peptide yield (ACH F1 & F6, FZH F1 & F6 and PEH F8) were selected for a second round of HPLC separation using the same C12 column with detection at 214 nm. The sample elution was carried out at 2 mL/min and the linear gradient and separation time was as follows: ACH F1 and F6 (23.50 – 50.0% for 35 min and 62.0 – 100.0% for 30 min, respectively, of mobile phase B); FZH F1 and F6 (19.1 – 50.0% for 30 min and 58.0 – 100.0% for 35 min, respectively, of mobile phase B); and PEH F8 (85.0 – 100.0% mobile phase B for 45 min). The pooled fractions from the second round of fractionation were prepared and analyzed for AChE inhibition in the same way as described for the first-round fractions and the most active fractions used for peptide identification by mass spectrometry.

### Mass spectrophotometry and identification of the peptide sequences

The mass spectrometer (MS) spectra were generated using an Absciex QTRAP^®^ 6500 System coupled with an electrospray ionization source (Absciex, Foster City, CA, USA) as previously described by Sonklin et al. (16) and Famuwagun et al. (17). Briefly, the freeze-dried samples from the second round of HPLC separation were each dissolved in 20% acetonitrile (50 μg/mL) and passed through a 0.2 μm filter. Subsequently, 10 μL of the filtered solution was directly infused into the mass spectrometer to obtain the MS. Some of the working conditions of the equipment were as follows: ion spray voltage (3.5 kV) at 150*^o^*C, and a flow rate of 30 μL/min for 3 min in the positive mode with maximum m/z scan set at 1,500 Da. The m/z values were used to identify peptide sequences from published primary structure of *Pisum sativum* proteins (with <± 0.001 Da mass tolerance) using the ExPASy Proteomics Server FindPept tool^1^, which was accessed in February 2022.

### Assay for the *in vitro* inhibition of acetylcholinesterase by the HPLC fractions and synthesized peptides

The Ellman assay (18) was performed as described by Balkis et al. (19) and Asen and Aluko (4) with some modifications. All the reagents used in the analysis were freshly prepared and stored in ice away from direct light to avoid degradation. Briefly, stock solutions of peptide or standard (galantamine) and their respective dilutions (10 – 50 μg/ml final concentration) were prepared in 0.1 M phosphate buffer (pH 7.4). The substrate ATCI (2.5 mM) was prepared in deionized water and the starting concentration for AChE was 5 U/ml prepared in the buffer. The reaction was carried out in a 96-well plate at a final volume of 300 μl containing ATCI (25 μl), AChE (25 μl), peptide sample or standard (25 μl), and 0.1 M phosphate buffer (pH 7.4) as A_sample._ The blank (A_blank_) reaction was carried out without the inhibitor to determine 100% of the enzymatic activity. The microplate was incubated in the plate reader at 37*^o^*C for 5 min after initial shaking. After incubation, 150 μl of freshly prepared DTNB (25 mM prepared in 50% ethanol) was added to all the wells and the absorbance (at 405 nm) was recorded for 10 min using a Powerwave XS2 microplate reader (Biotek instruments, Winooski, VT, USA) to create inhibition curves using the color production from DTNB and thiol complexes. The inhibition was calculated as follows:

Inhibition (%) = [(A_blank_ -A_sample_)/(A_blank_)] *100

The sample concentration that inhibited 50% AChE activity (IC_50_) was calculated by non-linear regression from a plot of peptide concentration versus percent inhibition.

### Kinetics of acetylcholinesterase inhibition activity by of the synthesized peptides and IC_50_ values

The kinetics of AChE inhibition by the peptides was determined as described by Fu et al. (20) with some slight modifications. Briefly, 10 and 50 μg/ml dilutions of the peptides were separately prepared from a stock solution in 0.1 M phosphate buffer (pH 7.4). Four substrate concentrations (0.156, 0.3125, 0.625, and 1.25 mM) were prepared from a stock solution of 10 mg/mL using deionized water and the final concentration of AChE was 0.5 U/ml prepared in the same buffer. The reaction was carried out in a 96-well plate at a final concentration of 300 μl containing ATCI (25 μl), AChE (25 μl), sample (25 μl), and 0.1 M phosphate buffer at pH 7.4 (A_sample_). The blank (A_blank_) was without the inhibitor to determine 100% of the enzymatic activity. The microplate was incubated in the plate reader at 37*^o^*C for 5 min after initial shaking. After incubation, 150 μl of freshly prepared DTNB (25 mM prepared in 50% ethanol) was added to all the wells and the absorbance (at 405 nm) was recorded for 10 min using a Powerwave XS2 microplate reader (Biotek instruments, Winooski, VT, USA) to create inhibition curves using the color production from DTNB and thiol complexes. The mode of AChE inhibition was estimated from Lineweaver-Burk plots and inhibition constant (Ki) was calculated as the *x*-axis intercept from a plot of the slope of the Lineweaver-Burk line versus peptide concentration prepared on GraphPad Prism 9.0 (GraphPad Software Inc., La Jolla, CA, USA).

### Intrinsic fluorescence emission of the acetylcholinesterase – Peptide complex

Intrinsic fluorescence spectra of the AChE – peptide complexes were measured as described by Fu et al. (20). Briefly, the 150 μl assay total volume contained AChE (200 μg/ml protein), mixed with one of three different peptide concentrations (1.56, 3.13, and 6.25 μg/ml), all prepared in 0.1 M phosphate buffer (pH 7.4). The emission spectra of the assay solutions were recorded at 25*^o^*C using a 100 μl capacity micro quartz cell in a fluorescence spectrophotometer Jasco FP-6300 (Jasco Corp., Tokyo, Japan). The assay solutions were excited at 280 nm and the emission spectrum obtained at 290 – 450 nm. The emission spectra of the buffer and peptides alone were subtracted from the respective emission spectrum to obtain the spectrum of each enzyme-peptide mixture.

### Circular dichroic spectra of the acetylcholinesterase–Peptide complex

The far and near UV spectra of the enzyme - peptide complexes were determined as described by Oluwagunwa et al. (21). The final assay solution (200 μl) was a mixture of enzyme (100 and 350 μg/ml for far and near-UV, respectively), peptide (12.5 – 50 μg/ml), which were all prepared in 0.1 M phosphate buffer (pH 7.4). A J-810 spectropolarimeter (Jasco Corp., Tokyo, Japan) was used to record the far-UV spectra at 190–240 nm with cuvette path length 0.05 cm and near-UV spectra at 250–320 nm with 0.1 cm cuvette for secondary and tertiary structures, respectively. The buffer spectrum was subtracted from the enzyme spectrum while the final enzyme-peptide complex spectra were obtained after deducting the respective peptide spectra. The secondary structures of the enzyme with or without the peptide complexes was analyzed by the deconvoluted far UV spectra using the SELCON3 algorithm, which was accessed through DichroWeb^2^ as described by Lobley et al. (22).

### Molecular docking analysis of acetylcholinesterase-peptide interaction for unraveling enzyme inhibition mechanism

Blind molecular docking of enzyme-peptide interaction was carried out with HPEPDOCK (23) by uploading the crystal structure of *Mus musculus* acetylcholinesterase retrieved from Protein Data Bank (PDB code: 2JF0, resolution: 2.50Å) and the various peptide sequences in the web server. Prior to docking, optimization of the crystal structure of the enzyme was performed with Chimera UCSF software version 1.15 (24) and Autodock vina package version 1.1.2 (25). This step involves structural energy minimization to reduce internal clashes, ignoring non-standard amino acid residues, elimination of solvents and complexed ligands crystalized with the enzyme, and addition of Gasteiger charges and polar hydrogen. The prepared crystal structure of AChE was uploaded in PDB format as receptor input whereas peptide sequence in FASTA format was entered as peptide input. MODPEP program in HPEPDOCK was used for refinement of peptide conformation (23). The docking result of the top model was analyzed with UCSF Chimera for charge environment, hydrophobicity/hydrophilicity regions, intermodal hydrogen bonding, and residues participating within 5Å to the binding sites.

### Statistical analysis

The experiments were carried out in triplicates and the data were expressed as mean ± standard deviation (SD). Analysis of variance (ANOVA) was used to determine the significant differences between mean values (*p* ≤ 0.05) by the Duncan’s multiple range test using IBM SPSS Statistics for Windows, Version 26.0.

## Results and discussion

### *In vitro* acetylcholinesterase inhibitory activity of peptides derived from pea protein

**FIGURE 1:**
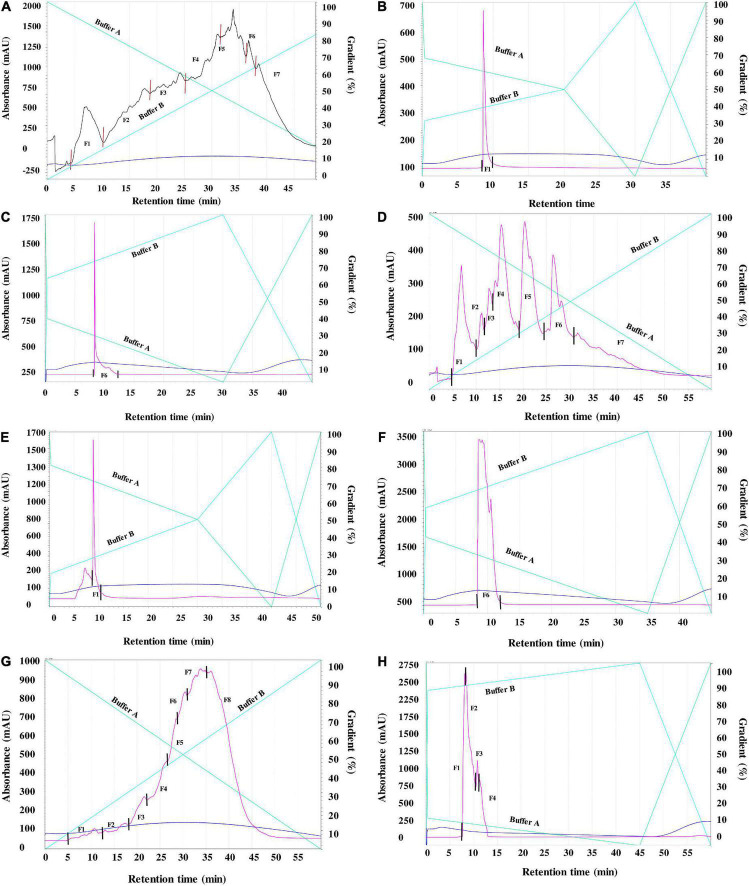
**(A)** First round (R1) RP-HPLC separation of peptides derived from enzymatic digests of pea protein using 4% alcalase (Fractions 1–7). **(B)**: Second round (R2) RP-HPLC separation of peptides derived from enzymatic digests of pea protein using 4% alcalase (Fractions 1). **(C)**: Second round (R2) RP-HPLC separation of peptides derived from enzymatic digests of pea protein using 4% alcalase (Fractions 6). **(D)**: First round (R1) RP-HPLC separation of peptides derived from enzymatic digests of pea protein using 4% flavourzyme (Fractions 1-7). **(E)**: Second round (R2) RP-HPLC separation of peptides derived from enzymatic digests of pea protein using 4% flavourzyme (Fractions 1). **(F)**: Second round (R2) RP-HPLC separation of peptides derived from enzymatic digests of pea protein using 4% flavourzyme (Fractions 6). **(G)**: First round (R1) RP-HPLC separation of peptides derived from enzymatic digests of pea protein using 1% pepsin (Fractions 1-8). Figure **(H)**: Second round (R2) RP-HPLC separation of peptides derived from enzymatic digests of pea protein using 1% pepsin Fraction 8 (1-4).

**FIGURE 2:**
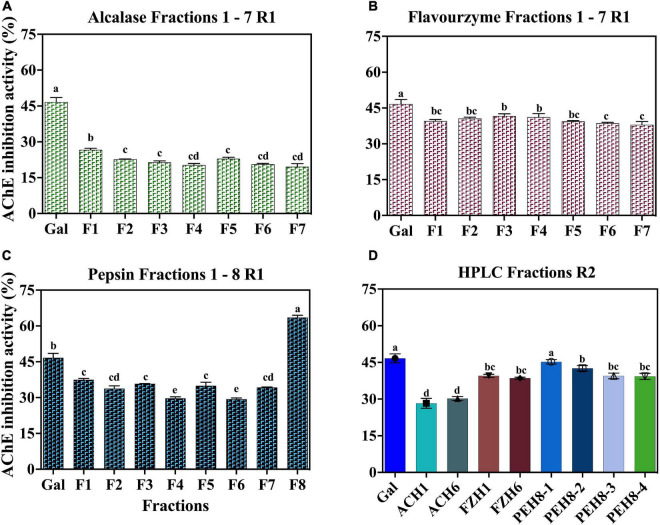
AChE-inhibitory activity of peptide fractions (30 μg) obtained after RP-HPLC separation of enzymatic digests of pea protein: First round **(A)** Alcalase **(B)** Flavourzyme **(C)** Pepsin and **(D)** Second round for all the hydrolysates.

Separation of the peptides present in ACH, FZH and PEH was carried using two consecutive reverse-phase HPLC fractionation rounds (Figures 1A-H). RP-HPLC purification is based on the changing levels of hydrophobicity where the less hydrophobic fractions are eluted earlier than the more hydrophobic. After the first round of separation, several HPLC fractions were obtained from ACH (F1-F7), FZH (F1-F7) and PEH (F1-F8) based on their retention time, and the initial AChE inhibition activities were determined (Figures 2A–C). The analysis was carried out at 10 - 50 μg/mL and the most potent activity was achieved at 30 μg/mL for all the samples. After the first round of separation, AChE inhibitory activity of FZH and some fractions of PEH was ∼50% greater than those ACH, and PEH F8 had stronger inhibitory activity than the positive control (*p* < 0.05). There was no significant difference in potency of the fractions obtained from ACH and FZH based on their elution time (*p* > 0.05), which suggests that the hydrophobicity of these fractions did not have strong influence on their potency but rather their peptide composition (9). On the other hand, the last fraction eluted from PEH (F8) was more potent than the other PEH fractions (*p* < 0.05) with AChE inhibitory activity >50%. A similar observation was reported about pepsin digest of hemp seed protein where eight HPLC fractions were obtained; however, F7 had the highest AChE inhibitory activity than the earlier-eluting fractions F1-F6 (9).

Low (2.5 mM) substrate (ATCI) level as described by Balkis et al. (19) was used in this work, when compared to the 15 mM used in our previous study (4). The reduced ATCI concentration improved the inhibitory activity of the peptides and the standard because at high substrate concentrations, the products of acetylcholine breakdown accumulate in the gorge within the active site, which prevent further substrate interactions with the enzyme (19). The substrate concentration used was low enough to reduce this type of substrate inhibition but at the same time high enough to detect strong inhibitory activity of the peptides (19). The AChE-inhibitory activity of the standard compound (galantamine) used in the current study improved by ∼10% above our previously published values where higher substrate concentration was used. After the first round of separation, the fractions for the second purification were selected based on their potency and peptide yield. The AChE-inhibitory activity of the selected fractions showed that only ACH had enhanced activity by ∼10% while there was no significant difference between the activities of FZH fractions (*p* > 0.05). However, the AChE-inhibitory activity of the four fractions pooled from PEH F8 declined by ∼25%, which shows that potency of the PEH F8 during the first separation could be due to the composition and synergistic interactions between different peptides. These observations could infer that optimal separation of FZH and PEH F8 was achieved during the first round (Figures 2A,B,D). This is contrary to a previous suggestion that bioactivity of peptides is enhanced by several cycles of purification through HPLC fractionation (26).

### *In vitro* acetylcholinesterase inhibitory activity of the synthesized peptides

Amino acid sequence of 20 oligopeptides and their locations within the primary structure of the yellow pea protein were identified as shown in Table 1. Out of the 20 identified peptides (4-7 amino acids in length), the majority were present in the PEH fractions (14 peptides) while FZH (4 peptides) and ACH (2 peptides) fractions contained the remainder. The identified peptides were synthesized *de novo* and their AChE–inhibitory IC_50_ values, shown in Table 1, revealed that the potency of the peptides (1.39 – 1.65 μg/ml) was not significantly different (*p* > 0.05) from the standard (1.60 μg/mL). The IC_50_ values of these peptides are lower than those reported for other peptide products in literature. For example, the AChE-inhibitory IC_50_ values of hemp seed protein hydrolysates were between 5.95 and 11.62 μg/*ml* (27) while Snakin-Z peptide isolated from *Ziziphus jujuba* fruit had IC_50_ value of 580 μg/ml (28). The use of plant metabolites in AD treatment is in commonplace and a study was carried out by Sierra et al. (29) using various concentrations (7.5 – 240 μg/ml) of alkaloids derived from *Zephyranthes carinata* herb to inhibit AChE activity. The results showed that the alkaloids had varying potencies (IC_50_ values of 1.96 – 53.63 μg/ml) and the standard had IC_50_ value of 0.59 μg/ml. Although the AChE-inhibitory potency of the standard in the referenced study is lower than what we have reported, the potency of the peptides from our current study is lower than that of the alkaloid galanthine (IC_50_ value = 1.96 μg/ml) as reported in the study (29). Another study by Frota et al. (30) characterized *in vitro* cholinesterase inhibition by phenolic compounds from a native medicinal plant in Northeastern Brazil (*Ouratea fieldingiana*) and two standards (physostigmine and galantamine) were used as the positive controls. The study reported that the IC_50_ of the phenolic compounds ranged between 3.12 μg/ml (apigenin) and 12.00 μg/ml (rutin). Another observation in the current study was that most of the peptides contained glutamine (Q) and those with slightly higher IC_50_ values (∼1.61-1.65 μg/ml) had no Q (i.e., HPVAINA, PLMLLA, VNRPGK, VNRFR, and LKSNDR), but the reason for this structural influence is not yet established. Consequently, QSQS, SQSRS, ETRSQ, PQDER, and LQHNA were selected for further tests as follows considering potency and shorter length. This is because smaller size peptides have better chance of being absorbed into the blood and go through the blood-brain-barrier than longer peptides.

**TABLE 1:** Peptide location within the primary structure of pea proteins and their inhibitory potency against acetylcholinesterase.

Peptide sequence**	IC_50_ (μg/mL)^1^	IC_50_ (μM)^2^	^†^Position	^††^Parent protein	^†††^Mother ions (Da)
SQSQ	1.58 ± 0.01^ab^	4.19	345-348	PEH Leg J	449.2
QSQS	1.60 ± 0.05^abc^	4.25	499-502	PEH Leg J	449.2
QHNAL	1.62 ± 0.02 ^abc^	3.29	76-80	FZH Leg A2	582.3
SGSDDN	n/a	n/a	506-511	FZH Cov	594.2
LQHNA	1.53 ± 0.02^ab^	3.10	75-79	FZH Leg A2	582.3
RSQSQ	1.57 ± 0.02^ab^	3.05	497-501	PEH Leg J	605.3
SQSRS	1.54 ± 0.02^ab^	3.26	119-123	PEH Leg J	605.3
ETRSQ	1.57 ± 0.02^ab^	2.96	258-262	PEH ProV	620.3
TRSQE	1.60 ± 0.03 ^abc^	3.00	259-263	PEH ProV	620.3
PQDER	1.54 ± 0.02^ab^	2.78	30-34	PEH Leg B	644.3
PLMLLA	1.61 ± 0.05 ^abc^	2.94	8-13	PEH Leg Vic	657.4
VNRPGK	1.61 ± 0.10 ^abc^	2.87	272-277	PEH Cov	670.4
VNRFR	1.61 ± 0.07 ^abc^	2.69	132-136	ACH Leg A, J,	691.4
QSHFAD	1.62 ± 0.07 ^abc^	2.71	434-439	PEH Vic	704.3
HPVAINA	1.65 ± 0.07 ^abc^	2.77	354-360	FZH Vic	721.4
QVFRAT	1.62 ± 0.04 ^abc^	2.64	460-465	ACH Leg J, k	721.4
DKKERG	1.54 ± 0.02^ab^	2.47	314-319	PEH Leg A	732.4
LKSNDR	1.61 ± 0.04 ^abc^	2.58	96-101	PEH Vic	732.4
KVSRDQ	1.39 ± 0.04^a^	2.22	213-218	PEH ProV	732.4
KNQKQS	1.44 ± 0.05^a^	2.30	537-542	PEH Vic	732.4
Galantamine***	1.60 ± 0.04 ^abc^	n/a	n/a	n/a	n/a

**Identified sequences of the peptides derived from pea protein digestion with 4% Alcalase, 4% Flavourzyme and 1% pepsin.

***An existing AD therapy used a positive control for Ellman’s assay.

^†^Peptide position in the mother chain.

^††^The globulin source of peptides.

^†††^m/zs of the peptide generated from mass spectrometer.

^1^IC_50_ values in μg/mL and ^2^IC_50_ values in μM.

Data are expressed as mean ± standard deviation (*n* = 3) and different letters (a-c) indicates significance difference at *p* < 0.05 mean values.

### Kinetics of acetylcholinesterase inhibition

**FIGURE 3:**
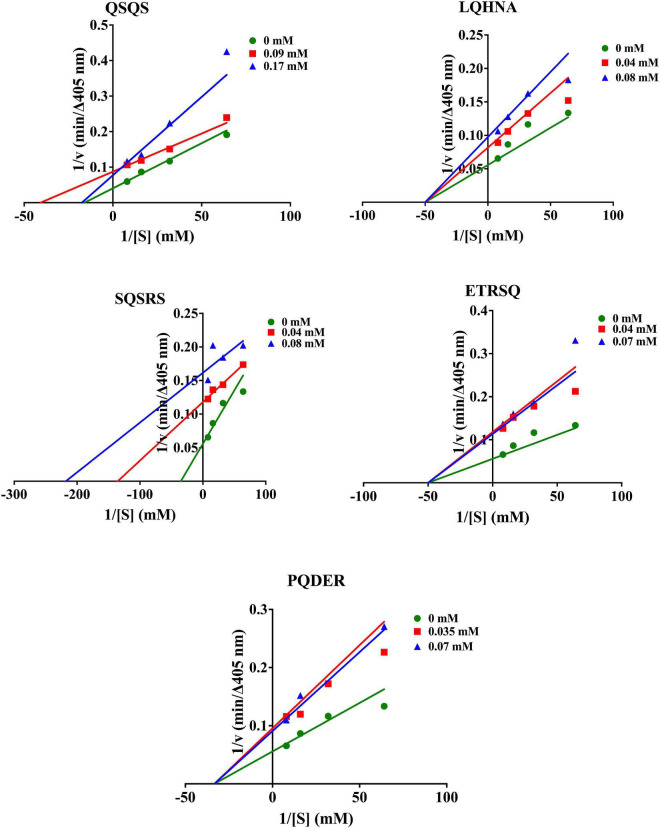
Lineweaver-Burk plots of AChE inhibition by the peptides.

As shown in Figure 3, Lineweaver-Burk plots were used to determine the type of AChE inhibition exhibited by QSQS, LQHNA, SQSRS, ETRSQ, and PQDER. The behavior of the peptides varied, and the AChE inhibition mode was dependent on the type of inhibitor. QSQS inhibited AChE by a mixed inhibition model, which was seen as a mixture of uncompetitive (parallel lines) and non-competitive (intersection at the *x*-axis) with decreased K_m_ and V_max_ values. Mixed inhibition suggests that the peptides could bind to both free enzyme and enzyme-substrate complex to reduce catalytic binding and hydrolysis of the substrate (9), which could also mean stronger inhibition than the other modes (11). LQHNA, ETRSQ, and PQDER showed non-competitive inhibition, which was evident as intersection of lines at the *x*-axis but different intersections at the *y*-axis. In this model, the K_m_ remains similar while V_max_ decreased in the presence of the peptides (Table 2). Non-competitive inhibition means the peptide binds to a non-active site in an enzyme-substrate complex or free enzyme to reduce the catalytic activity (31–35). SQSRS inhibitory effect was uncompetitive, as indicated by the parallel lines, and this means that the peptide will not compete for the binding site with the natural substrate but will bind to the enzyme-substrate complex and prevent conversion of the substrate to products (8, 32). Different inhibition modes have been recorded in literature for peptides and phenolic compounds used as AChE inhibitors (8, 9, 33–35). There is a contrast between the catalytic parameters of AChE inhibition by hemp seed peptides (V_max_ 0.0036 - 0.025 mM; K_m_ 0.026 – 0.66 mM/min; Ki 0.014 – 0.025 mg/ml) as reported by Malomo and Aluko (9) and the kinetic values obtained for peptides in the current study. This shows that the AChE-inhibitory activity of peptides is largely dependent on the peptide sequence, which is determined by the source of the protein and the proteases used in the digestion. The inhibition constant was relatively low (K_i_ < 0.20 mM) for most peptides in the current study except QSQS that had *K*_i_ = 0.43 mM. The V_max_ reduced in the presence of this peptide and the low K_m_ values (<0.1 mM) suggest strong affinity for AChE. Low K_i_ values indicate that only small peptide concentrations are required to inhibit AChE activity, which is evident in the strong potency of most of the peptides even at low concentrations. The increased K_m_ values indicate that peptide binding to AChE prevented normal formation of AChE-ACh complex or conversion of ACh into products (20, 36).

**TABLE 2:** Kinetics constants of AChE catalyzed reaction at different peptide concentrations.

		*QSQS		*LQHNA		*SQSRS		*ETRSQ		*PQDER	
^†^Catalytic parameter	Control	0.1 mM	0.05 mM	0.1 mM	0.05 mM	0.1 mM	0.05 mM	0.1 mM	0.05 mM	0.1 mM	0.05 mM
Vmax (ΔA/min)	17.96 ± 0.01^a^	11.46 ± 0.00^bc^	12.99 ± 00^b^	12.25 ± 0.00^b^	10.27 ± 0.00^c^	8.50 ± 0.00^d^	6.20 ± 0.00^e^	8.83 ± 0.00^d^	8.50 ± 0.00^d^	11.03 ± 0.00^c^	10.48 ± 0.00^c^
Km (mM)	0.03 ± 0.00^b^	0.02 ± 0.00^b^	0.06 ± 0.00^a^	0.02 ± 0.00^b^	0.02 ± 0.00^b^	0.01 ± 0.00^b^	0.01 ± 0.00^b^	0.01 ± 0.00^b^	0.01 ± 0.00^b^	0.02 ± 0.00^b^	0.02 ± 0.00^b^
Ki (mM)		0.43		0.16		0.02		0.06		0.08	

*Selected peptides for the analysis at 0.1 and 0.05 mM.

^†^*V*_max_ is the maximum velocity, *K*_m_ is the Michaelis constant, and *K*_i_ is the enzyme-inhibitor dissociation constant.

Data are expressed as mean ± standard deviation (*n* = 3) and different letters (a-d) indicates significance difference at *p* < 0.05 mean values in the columns.

### Intrinsic fluorescence spectra of acetylcholinesterase and fluorescence quenching by the peptides

**FIGURE 4:**
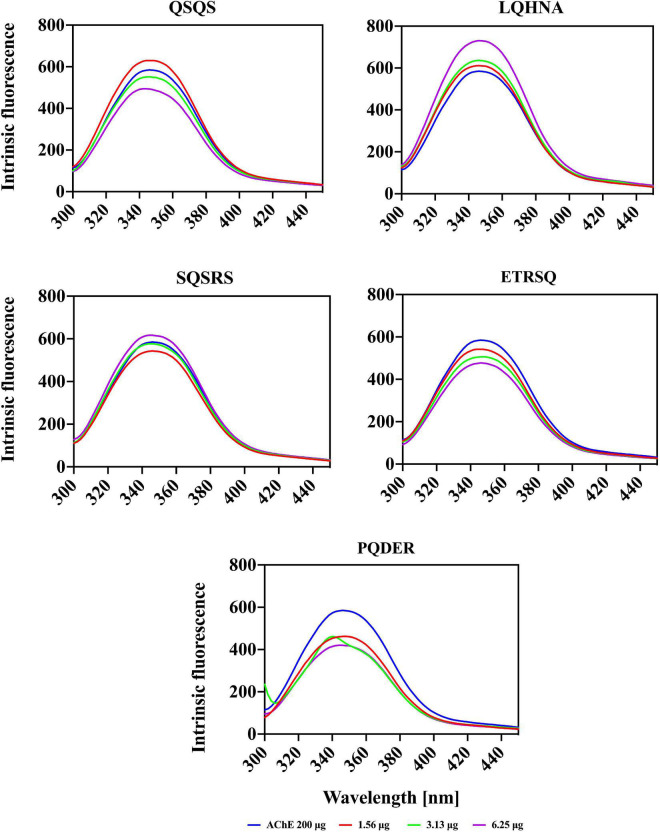
Fluorescence intensity spectra of AChE in the absence and presence of peptides.

Conformational changes in AChE protein structure upon interaction with the different peptide concentrations was analyzed using intrinsic fluorescence intensity (FI) spectra. The maximum FI of a protein is directly related to the physicochemical structure of the three fluorophore amino acids namely tryptophan (*Trp*), tyrosine (*Tyr*) and phenylalanine (*Phe*) in addition to polarity of the microenvironment (37). However, due to the presence of an indole group, *Trp* has the longest wavelength for excitation and emission spectra (∼ 280-295 and 350 nm, respectively) and a longer lifespan, hence higher contributions to FI than *Tyr* and *Phe*. The excitation and emission spectra of AChE alone and in the presence of peptides are shown in Figure 4. The maximal emission spectrum of the AChE was achieved at 342 nm, which is a characteristic of *Trp* residues in a slightly hydrophilic environment (longer wavelength or red shift) and infers that there was some degree of structural unfolding of the enzyme in the aqueous solution (37, 38). A small peak was observed at 291 nm, which could be the presence of *Phe* residues in a hydrophilic environment. Interactions between the enzyme and different peptide concentrations produced varying quenching effects, which were dependent on the type of peptide and concentration. Increasing the concentration of QSQS (except at 1.56 μg/ml), SQSRS (except at 6.25 μg/ml), ETRSQ and PQDER reduced the FI of the enzyme, which depicts a less compact structure due to increased distances between the fluorophore molecules as well as their greater interactions with the hydrophilic environment that results in fluorescence quenching. However, the structure of the enzyme became more compact after interaction with LQHNA, and a higher intensity occurred with the *Phe* residue at 3.13 μg/ml peptide concentration. A study carried out by Fale et al. (15) to determine the interaction between *Plectranthus barbatus* herbal tea components and AChE showed that only *Trp* residues contributed to the fluorescence emission of the enzyme. The study also showed that there was no shift in the position of the *Trp* residues, which means that although the inhibitor interacted very closely with AChE to quench fluorescence, the secondary and tertiary structures of the enzyme may not have been altered (15). Another study showed that increasing the concentration of polyphenols from *Phyllanthus emblica Linn* fruit in interaction with AChE reduced the FI and produced a blue (quercetin and fisetin) or red (gallic acid) shift, which was dependent on the substances involved (35). Reduced fluorescence emission confirms interaction of the peptides with the enzyme and, although there was no structural unfolding detected for AChE after exposure to LQHNA, the low Ki value of this complex showed there was good binding affinity (20). Bai et al. (35) reported that inhibitory activity and catalytic properties of inhibitors against enzymes do not have a strict correlation with fluorescence properties because emission depends on the interaction between the inhibitors and fluorophores or the microenvironment. Therefore, for some inhibitors, fluorescence emission can only confirm the interactions with the enzyme but not potency of the disruption of catalytic activity.

### Circular dichroic spectra of acetylcholinesterase and effects of the peptides

**FIGURE 5:**
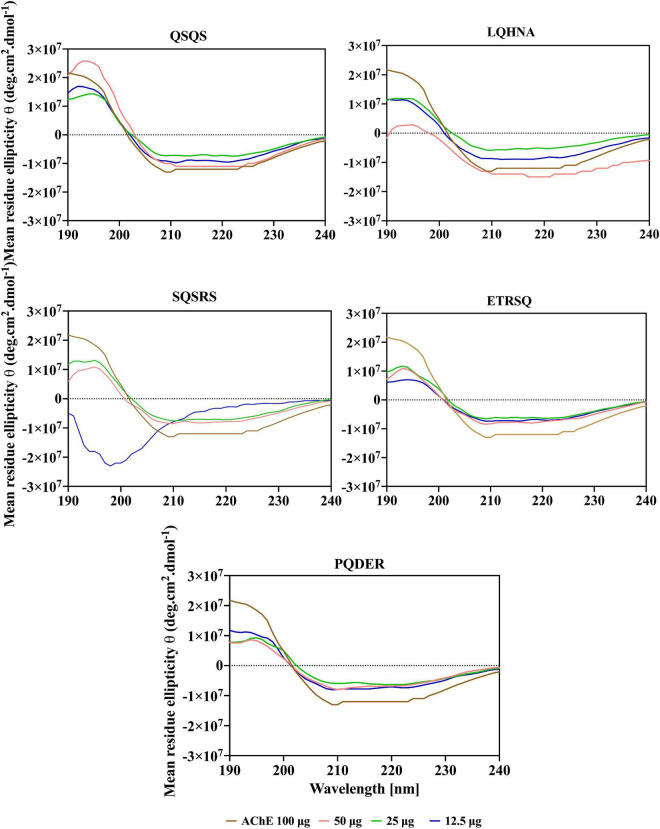
Far-UV spectra of AChE in the presence of different concentrations of peptides.

**FIGURE 6:**
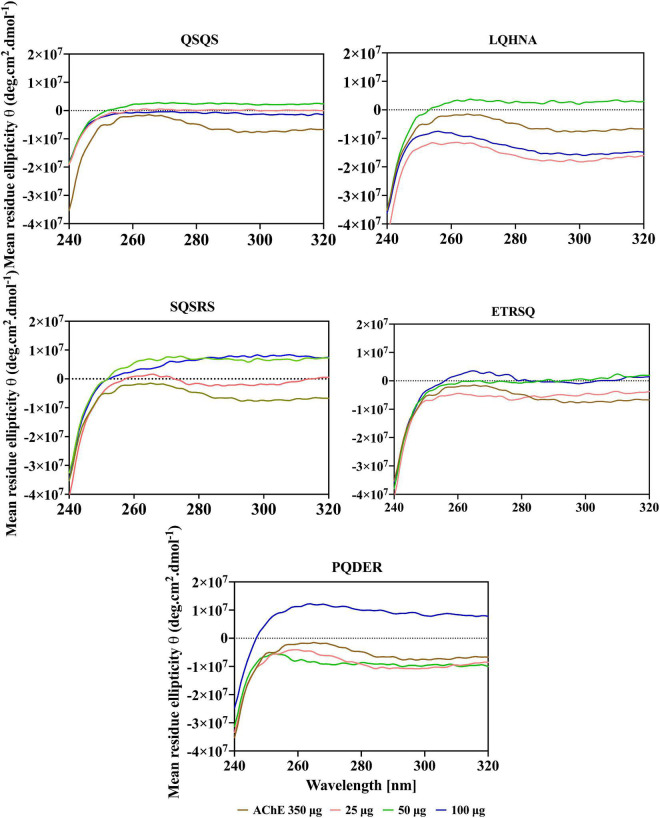
Near-UV spectra of AChE in the presence of different concentrations of peptides.

Additional evidence for interaction the between AChE and the peptides was obtained from far and near-UV Circular dichroic (CD) spectra of the enzyme in an aqueous solution with or without the inhibitor. CD analyzes the protein structure based on differences in the right and left circularly polarized light (37). As shown in Figures 5, 6 and Table 3, the native enzyme showed preponderance of α- helical structures (36%), β-sheets and strands (16% each) and unordered structures (31%). This was seen as a positive peak at 191 nm, zero crossing at 202 nm and other negative peaks at 209 and 219 nm (39). Similarly, the high degree of unordered structures could reflect an open active site structure as shown by the degree of unfolding of AChE structure from the intrinsic fluorescence spectra in Figure 4. A previous work reported the FTIR spectra of AChE with similar result of 35% α-helical and 16% β-sheet fractions (38). AChE has high conformational flexibility due to its spatial arrangement of a pair of four helical structures placed in an antiparallel fashion leaving a large space at the center (15). As a result, the enzyme has increased solvation in aqueous environments, which could be the explanation for the slightly unfolded structure of AChE as observed with the fluorescence spectra (38). The far-UV CD spectra of AChE showed that structural changes in the presence of inhibitors were dependent on peptide type and concentration and, for most of the peptides, the helical structure was significantly decreased with a concomitant increase in the β-sheets (*p* ≤ 0.05). In Figure 5, a red shift in the far-UV of the enzyme occurred after the addition of LQHNA, SQSRS, ETRSQ, and PQDER, especially at 50 μg/mL peptide concentration. Increase in ellipticity values characterizes the loss of helical structure and increase in the β-sheets (40). The structural changes of the enzyme were minimal after interaction with QSQS, and a slight blue shift occurred at the zero-line crossing with a small increase in ellipticity values. A negative ellipticity occurred after the interaction of AChE with low concentration of SQSRS producing mostly ordered structures in the enzyme seen as a large negative peak at 197 nm. These observations suggest high inhibitory activity of this peptide as evidenced by the low V_max_ and K_i_ values (Table 2).

**TABLE 3:** Estimated secondary structure composition of AChE and AChE-peptide complex.

^†^Elements	AChE*	QSQS**	LQHNA**	SQSRS**	ETRSQ**	PQDER**
**α -Helix**	**35.9 ± 0.05^a^**					
0.0125		29.95 ± 0.00^b^	24.70 ± 0.02^c^	3.70 ± 0.00^e^	19.65 ± 0.02^d^	22.55 ± 0.00^c^
0.025		24.25 ± 0.01^c^	19.05 ± 0.01^d^	21.95 ± 0.01^c^	22.00 ± 0.01^c^	20.35 ± 0.01^c^
0.05		37.50 ± 0.05^a^	0.01 ± 0.00^f^	23.75 ± 0.01^c^	23.50 ± 0.01^c^	20.45 ± 0.01^c^
**β -Strands**	**16.30 ± 0.08^e^**					
0.0125		19.90 ± 0.01^de^	22.90 ± 0.01^cd^	14.40 ± 0.01^e^	24.30 ± 0.01^c^	25.55 ± 0.01^c^
0.025		23.75 ± 0.00^d^	74.70 ± 0.19^b^	27.22 ± 0.01^c^	26.70 ± 0.02^c^	26.90 ± 0.02^c^
0.05		18.40 ± 0.02^de^	87.40 ± 0.18^a^	22.01 ± 0.02^cd^	22.50 ± 0.03^cd^	25.15 ± 0.02^c^
**β -Turns**	**16.20 ± 0.02^ab^**					
0.0125		18.30 ± 0.03^a^	8.25 ± 0.01^c^	19.55 ± 0.02^a^	19.70 ± 0.02^a^	19.05 ± 0.01^a^
0.025		18.95 ± 0.02^a^	16.50 ± 0.01^ab^	19.45 ± 0.02^a^	19.00 ± 0.01^a^	17.90 ± 0.01^a^
0.05		19.30 ± 0.02^a^	19.25 ± 0.03^a^	10.50 ± 0.02^c^	18.50 ± 0.02^a^	18.70 ± 0.02^a^
**Unordered structure**	**31.60 ± 0.01^bc^**					
0.0125		30.85 ± 0.01^bc^	33.15 ± 0.04^bc^	71.35 ± 0.03^a^	37.50 ± 0.02^b^	33.20 ± 0.03^bc^
0.025		33.05 ± 0.01^bc^	31.50 ± 0.00^bc^	31.35 ± 0.01^bc^	32.20 ± 0.02^bc^	35.40 ± 0.03^b^
0.05		26.99 ± 0.01^c^	4.40 ± 0.06^d^	34.65 ± 0.03^b^	34.25 ± 0.03^b^	34.90 ± 0.02^b^

*Secondary structures of the AChE and**selected peptides.

^†^Secondary elements of the enzyme with or without interaction with the peptides.

Data are expressed as mean ± standard deviation (*n* = 3) and different letters (a-f) indicates significance difference at p < 0.05 mean values in the columns.

The CD spectrum in near-UV region (240 – 320 nm) gives information about the tertiary structure of the enzyme and it is a contribution from the aromatic amino acids and disulfide linkages (37). The results (Figure 6) show that the near-UV signals of native AChE were low, which indicates an unfolded and less defined structure that is consistent with data from the fluorescence emission spectra in Figure 4. The ellipticity value of the enzyme increased slightly after concentration dependent interaction with QSQS, ETRSQ and SQSRS while changes in the presence of different concentrations of LQHNA and PQDER had no defined order (Figure 6). Therefore, the peptides influenced changes in AChE protein conformation to varying degrees. Slight increase in ellipticity values of the enzyme is due to the formation of a more compact structure allowing for stronger interactions between the chromophores while lower values mean that the chromophores experience some level of signal quenching because of interactions with the hydrophilic environment (41).

### Molecular docking of the peptides to acetylcholinesterase

**FIGURE 7:**
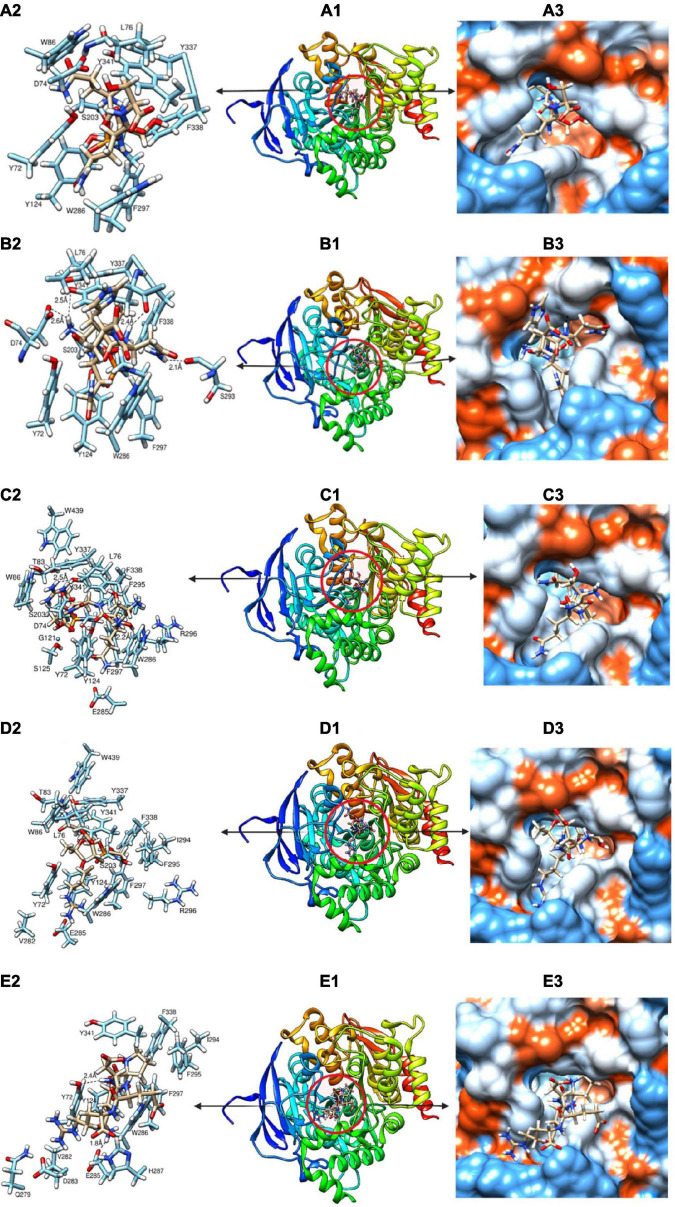
**(A-E)** Molecular docking models showing the binding pattern of QSQS **(A1)**, LQHNA **(B1)**, SQSRS **(C1)**, ETRSQ **(D1)** and PQDER **(E1)** to the active site of acetylcholinesterase. Amino acids interacting within 3Å **(A2, B2, C2, D2, and E2)** and Kyte-Doolittle scale **(A3, B3, C3, D3, and E3)** depicting charge environment and hydrophilicity/hydrophobicity of the binding site with colors ranging from dodger blue for the most hydrophilic to white 0.0 to orange red for the most hydrophobic.

The mechanism of inhibition of AChE by the various peptides was investigated by molecular docking using a high precision docking program, HPEPDOCK, and the results presented in Table 4 and Figures 7A–E. The docking energy score obtained from HPEPDOCK is a measure of relative binding affinity and stability of peptide–AChE complexes. It uses the SIMPLEX minimization algorithm binding energy scores particularly programmed for protein–protein and protein–peptide interactions. This docking tool is different from other commonly used programs because it employs iterative knowledge-based scoring function for protein-peptide and protein-protein interactions rather than the original scoring function used for protein–ligand interactions (23, 42). The docking energy scores are calculated from the total contribution of various intermolecular interactions such as hydrophobic, van der Waals forces, electrostatic, hydrogen bonding, conformational state, and entropy of the peptide, in the binding of the peptide and stabilization of the complex (43). The binding affinity of the peptide to AChE was measured by the extent of the negative docking energy scores, the higher the negative value, the greater the affinity and stability of the complex formed and shows that physiological response could be triggered by low peptide dose. The binding energy scores of the five peptides ranged from −123.5 to −164.5 with LQHNA, SQSRS and PQDER having the highest affinity, and QSQS and ETRSQ showing the lowest binding strength. The results suggest important contributions of the presence of glutamine at the penultimate N-terminal position (second position) in enhancing affinity between peptides and AChE. All the peptides docked in between the peripheral anionic site (PAS) and CAS of AChE, which are consistent with the mixed, non-competitive, and uncompetitive inhibition modes obtained from the enzyme inhibition kinetics data in Figure 5. The peptides interacted with all the amino acid residues of the PAS (Y72, D74, Y124, W286, and Y341) and at least one of the three amino acid residues that form the catalytic triad (S203, E334, and H447), which indicate ability to compete with the substrate (44). For instance, QSQS, LQHNA, and ETRSQ were bound near S203 and H447, while SQSRS and PQDER interacted with H447 and S203, respectively (Table 4 and Figure 7). Peptide binding in the region of these two major binding sites of AChE with moderate negative docking energy score, strong hydrogen bonding pattern and spontaneous formation of stable peptide-AChE complexes could be responsible for the observed strong inhibitory effect on the enzyme activity (IC_50_ ∼ 1.53 – 1.60 μg/ml). Therefore, the results suggest that the mechanism of AChE inhibition by these peptides is through binding to the enzyme CAS (which is responsible for ACh hydrolysis) and the PAS (responsible for non-cholinergic function) (45), and thus can be classified as bimodal. LQHNA, SQSRS and PQDER undertook a total of strong 4, 2, and 2 hydrogen bonding and formed 3, 1, and 1 hydrogen bonding, respectively with the three amino acids of the PAS at bonding distances in the range of 1.8 to 2.6 Å (Table 4 and Figure 7). LQHNA (IC_50_ = 1.53 μg/ml) having the highest number of hydrogen bonding including those formed with the amino acid residues of the PAS showed the highest affinity whereas QSQS and ETRSQ (IC_50_ = 1.57 and 1.60 μg/ml, respectively), which demonstrated the lowest binding affinity, did not show any hydrogen bond pattern. The peptides had similar IC_50_ values but the K_i_ value of QSQS was the highest among the peptides (Table 2), which could be an explanation for the lowest binding affinity of the peptide. This is an indication that hydrogen bonding could be playing essential role in the interaction and stability of the peptide–AChE complexes. Furthermore, glutamine (Q) is present in all the peptides, but the common feature of the higher binding peptides (LQHNA, SQSRS, and PQDER) is the position of Q in the tetrapeptide C-terminal sequence, which may have contributed to the higher binding affinity and resulted in an optimal peptide conformation that induced stronger interaction with AChE when compared to the interactions formed by QSQS and ETRSQ. The Kyte-Doolittle model (Figure 7) showed that the peptides generally underwent amphipathic interaction with the amino acid residues of the gorge.

**TABLE 4:** Relative binding affinity of peptide to acetylcholinesterase, residues involved within 5Å to the binding site, and hydrogen-bonding pattern.

	AChE – Peptide binding				
No.	Peptide sequence	Docking energy scores	Residues involved within 5 Å	No. of H-bonding	H-bond distance
2	QSQS	−123.524	Y72, V73, D74, T75, L76, G82, T83, W86, N87, G121, G122, Y124, S203, S125, V282, E285, W286, I294, F295, R296, F297, S298, Y337, F338, V340, Y341, G342, W439, H447	0	-
4	L**QHN**A	−164.517	Y72, D74 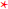 , T75, L76, T83, W86, G121, G122, Y124, S125, S203, E285, W286, H287, L289, E292, S293 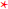 , I294, F295, F297, I365, Y337, F338, L339, V340, Y341 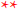 , G342, H447	4	2.1-2.6
7	SQS*R*S	−155.921	Y72, V73, D74, T75, G82, T83, E84, M85, W86, N87, G120, G121, G122, Y124, S125, G126, V282, E285, W286, L289, S293, I294, F295, R296 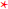 , F297, Y337, F338, Y341 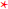 , G342, W439, H447, G448	2	2.2, 2.5
8	ETRSQ	−148.779	Y72, D74, T75, L76, G82, T83, M85, W86, N87, G121, G122, Y124, S203, V282, D283, H284, E285, W286, H287, S293, I294, F295, R296, F297, S298, F299, Y337, F338, Y341, G342, W439, H447, Y449	0	-
10	PQ*D*ER	−160.568	Y72 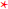 , V73, D74, T75, L76, G122, Y124, G154, S203, Q279, V282, D283, H284, E285 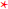 , W286, H287, L289, E292, S293, I294, F295, R296, F297, S298, F299, Y337, F338, Y341, G342	2	1.8, 2.4

*Indicates H-bond donor which could be *, **, *** for 1, 2, 3 donors, respectively.

*Indicates H-bonds acceptor which could be 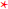, 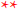, 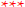 for 1, 2, 3 acceptors respectively.

*Binding sites*: Residues forming the catalytic active site (CAS) triad: S203, E334, H447 (6, 38). Residues forming the peripheral anionic site (PAS): Y72, D74, Y124, W286, Y341 (6, 38).

## Conclusion

This study determined conformational changes, kinetics of AChE inhibition and potential enzyme-ligand binding configurations of five novel yellow field pea protein-derived peptides that exhibited potency as AChE inhibitors. The five peptides analyzed had similar IC_50_ values and relatively low *Ki* values, and they exhibited different types of reversible inhibition models (i.e., non-competitive, uncompetitive, and mixed). The inhibitory mode was confirmed by molecular docking analysis, which revealed that the peptides acted through bimodal binding to both the CAS and PAS. On interaction with peptides, there was a concentration-dependent decrease in AChE fluorescence intensity, except for LQHNA. The CD spectra analysis showed that AChE had a high content of helical structure, which was significantly reduced in the presence of the peptides. The near-UV spectra indicated a relatively unfolded conformation of the native AChE in aqueous solution, which was modified by the peptides. However, information from the conformational changes had no direct relationship with peptide potency and catalytic parameters but the binding affinity of the peptides from molecular docking analysis corresponded with the AChE-inhibitory potency. The presence of glutamine (Q) in the five potent peptides analyzed is an important discovery with respect to structural requirements of AChE-inhibitory peptides. However, more studies will be needed to establish the role of the number and position of glutamine and other amino acid residues in enhancing potency of AChE inhibiting peptides.

## Data availability statement

The original contributions presented in this study are included in the article/Supplementary materials, further inquiries can be directed to the corresponding author.

## Author contributions

RA: conceptualization. NA and OO: analysis, investigation, and original draft. RA: funding. RA and CU: supervision and review and editing. All authors contributed to the article and approved the submitted version.

## Footnotes

1
http://web.expasy.org/findpept

2
http://dichroweb.cryst.bbk.ac.uk/html/home.shtml
