# Supplementary figures and images for: In vitro inhibition of acetylcholinesterase activity by yellow field pea (Pisum sativum) protein-derived peptides as revealed by kinetics and molecular docking
Source: Front Nutr. 2022 Oct 21;9:1021893. doi: 10.3389/fnut.2022.1021893 (PMC9635817; doi:10.3389/fnut.2022.1021893)

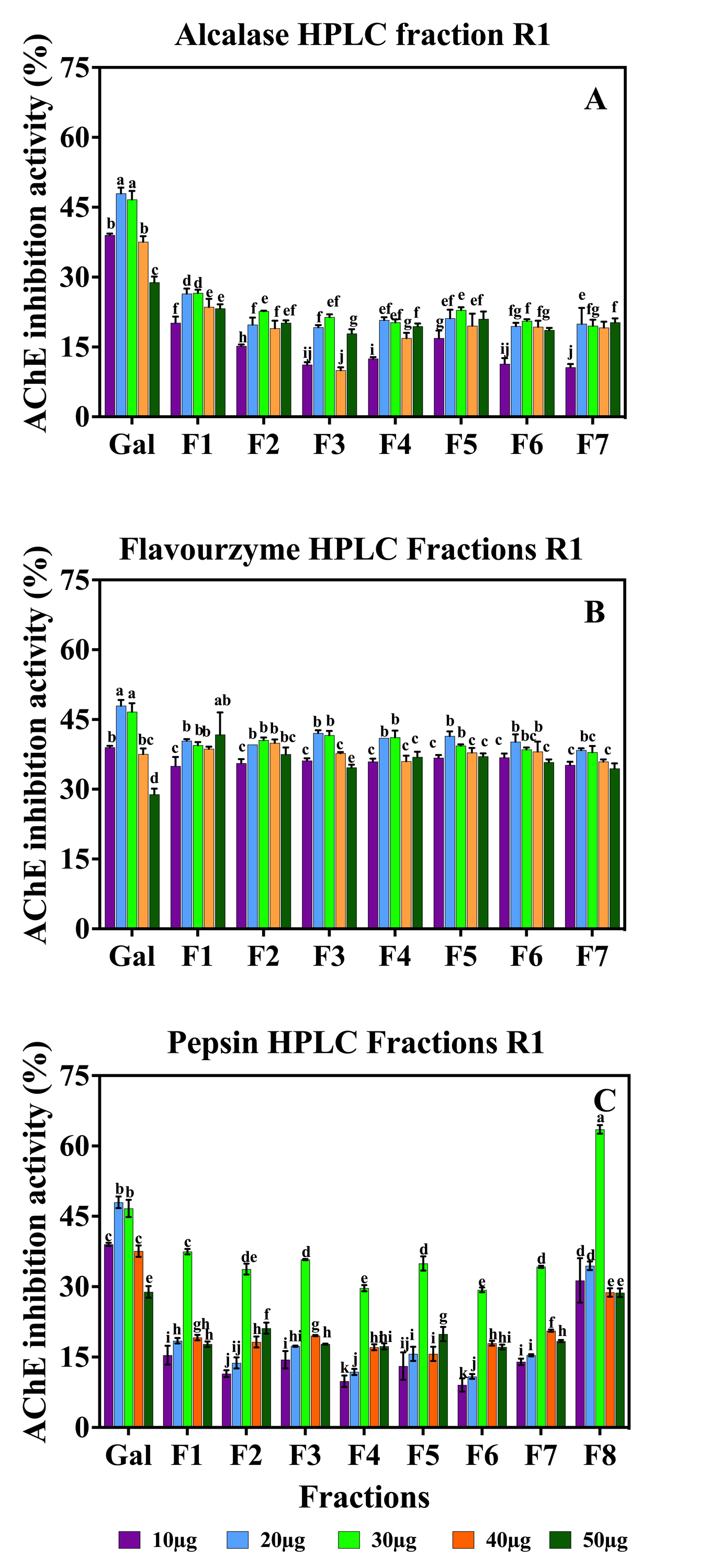

Supplement: Supplementary file 1 [file Image_1.JPEG]

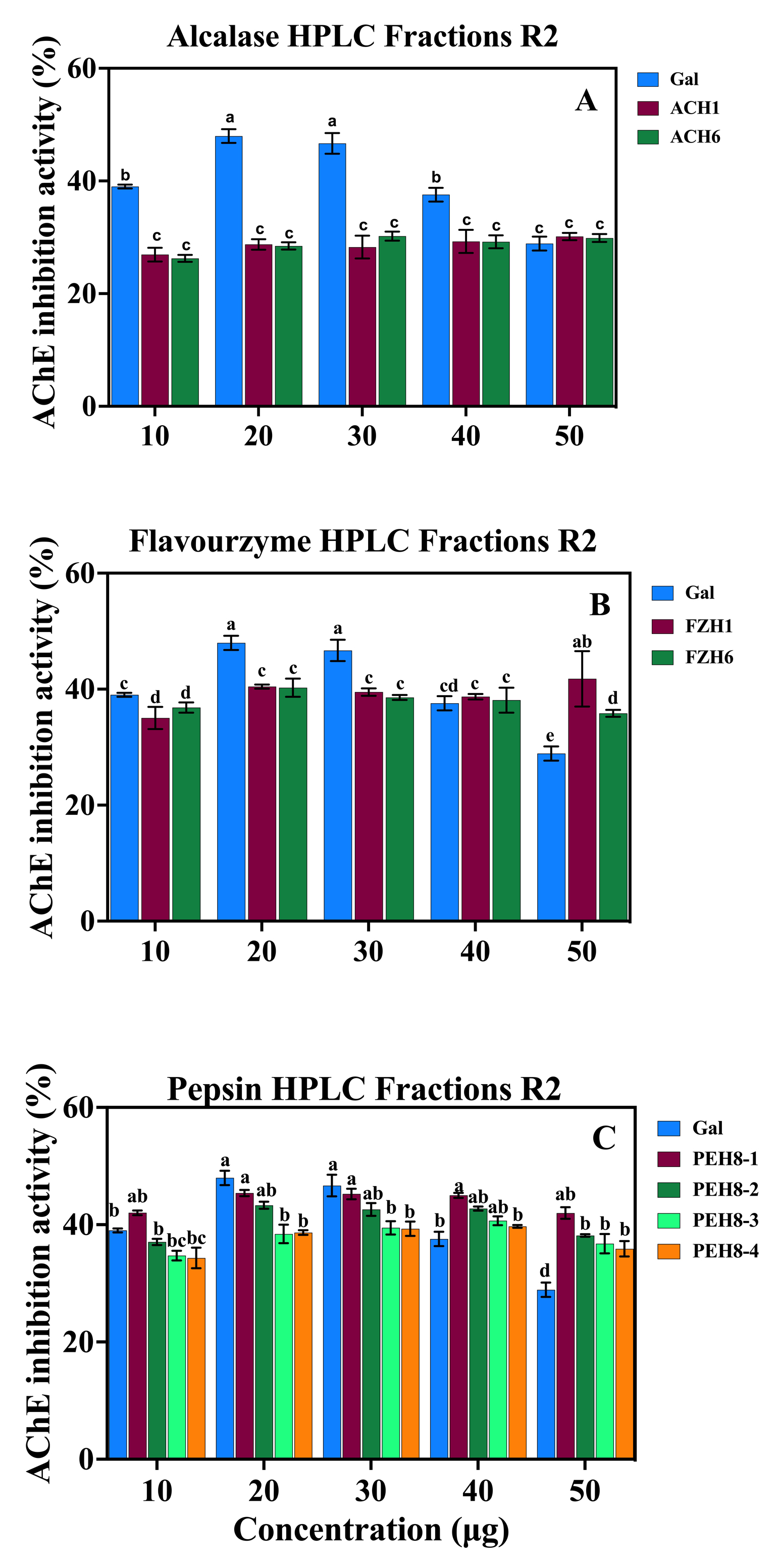

Supplement: Supplementary file 2 [file Image_2.JPEG]
